# Effects of calreticulin mutations on cell transformation and immunity

**DOI:** 10.1111/jcmm.17713

**Published:** 2023-03-13

**Authors:** Harini Desikan, Amanpreet Kaur, Irina D. Pogozheva, Malini Raghavan

**Affiliations:** ^1^ Department of Microbiology and Immunology University of Michigan Medical School Ann Arbor Michigan USA; ^2^ Department of Medicinal Chemistry College of Pharmacy, University of Michigan Ann Arbor Michigan USA

**Keywords:** calcium, calreticulin, endoplasmic reticulum, MHC Class I, MPL, myeloproliferative neoplasms, thrombopoietin receptor

## Abstract

Myeloproliferative neoplasms (MPNs) are cancers involving dysregulated production and function of myeloid lineage hematopoietic cells. Among MPNs, Essential thrombocythemia (ET), Polycythemia Vera (PV) and Myelofibrosis (MF), are driven by mutations that activate the JAK–STAT signalling pathway. Somatic mutations of calreticulin (CRT), an endoplasmic reticulum (ER)‐localized lectin chaperone, are driver mutations in approximately 25% of ET and 35% of MF patients. The MPN‐linked mutant CRT proteins have novel frameshifted carboxy‐domain sequences and lack an ER retention motif, resulting in their secretion. Wild type CRT is a regulator of ER calcium homeostasis and plays a key role in the assembly of major histocompatibility complex (MHC) class I molecules, which are the ligands for antigen receptors of CD8^+^ T cells. Mutant CRT‐linked oncogenesis results from the dysregulation of calcium signalling in cells and the formation of stable complexes of mutant CRT with myeloproliferative leukemia (MPL) protein, followed by downstream activation of the JAK–STAT signalling pathway. The intricate participation of CRT in ER protein folding, calcium homeostasis and immunity suggests the involvement of multiple mechanisms of mutant CRT‐linked oncogenesis. In this review, we highlight recent findings related to the role of MPN‐linked CRT mutations in the dysregulation of calcium homeostasis, MPL activation and immunity.

## 1 CALRETICULIN (CRT) MUTATIONS IN CANCER

Myeloproliferative Neoplasms (MPNs), including Polycythemia Vera (PV), Essential thrombocythemia (ET) and primary Myelofibrosis (MF), are a group of clonal hematopoietic stem cell (HSC) disorders characterized by the increased production of myeloid lineage cells.^1^ PV is marked by an overproduction of red blood cells, which is often accompanied by thrombocytosis and/or leukocytosis. ET is associated with increased platelet counts. MF is characterized by bone marrow (BM) hypercellularity, scarring of the BM due to reticulin or collagen fibrosis, and extramedullary hematopoiesis.^2^ While the majority of MPN patients have mutations in the Janus Kinase 2 tyrosine kinase gene (*JAK2*), a substantial proportion of ET and MF patients with non‐mutated *JAK2* have mutations in exon 9 of the CRT gene (*CALR*). A small percentage of ET and MF patients carry mutations in the gene encoding the thrombopoietin receptor (TPOR), also known as the myeloproliferative leukemia protein (MPL).^3^, ^4^ While the most common *JAK2* V617F mutation is found in around 95% of PV patients and 60% of ET and MF patients, about 15%–25% of ET and 25%–36% of MF patients carry *CALR* mutations, and 4%–9% of ET and MF patients carry *MPL* mutations.^3^, ^4^, ^5^, ^6^

**FIGURE 1:**
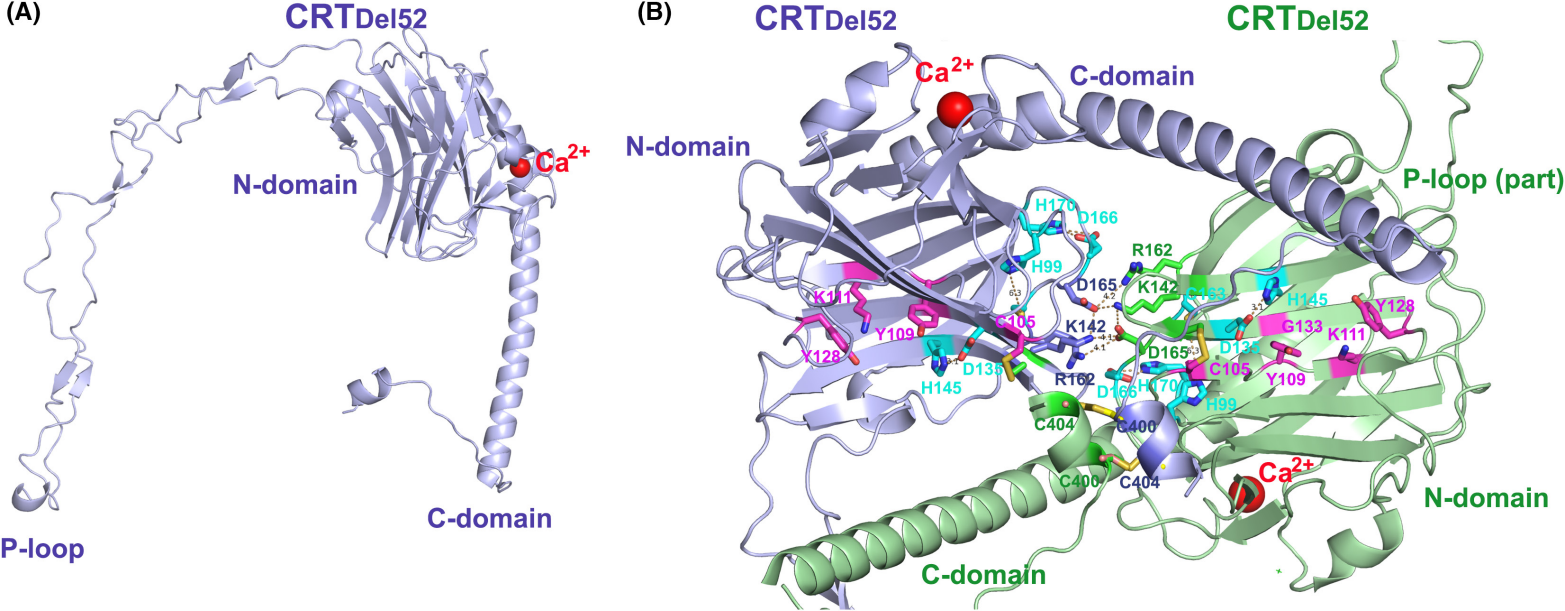
Structural models of CRT_Del52_ monomer (A) and dimer (B). The CRT monomer structure was generated by AlphaFold.^90^ The dimer was modelled using the crystal structure of human CRT D71K^91^ (PDB ID: 5LK5, subunits G and E) as described earlier.^52^ Residues forming ionic pairs at the dimerization interfaces (K142, R162, D165) are shown as blue and green sticks for the two subunits. Residues forming the glycan binding site (C105, Y109, K111, Y128 and G133)^12^ are shown as purple sticks. Residues suggested to be involved in Zn^2+^‐binding (H99‐C163, H145‐D135 and H170‐D166)^54^ are shown as cyan sticks. A Ca^2+^ ion in the high‐affinity site of each monomer is shown as a red sphere. Figures 1, 2, 4 and 5 were prepared using the PyMOL Molecular Graphics System (version 1.8.4.2) Schrödinger, LLC.

CRT is an ER‐resident calcium‐binding chaperone that assists in the folding of N‐glycosylated proteins.^7^, ^8^, ^9^, ^10^, ^11^ Essential to this function is a glycan‐binding site located in the amino‐terminal domain (N‐domain; Figure 1A) that interacts with asparagine‐linked (N‐linked) monoglucosylated glycans of glycoproteins.^12^ Over 50 *CALR* mutations have been linked to MPNs, with the two most frequent being a 52 base pair deletion (CRT_Del52_) and a 5 base pair insertion (CRT_Ins5_).^3^, ^4^, ^13^ All the mutations cause a frameshift in the carboxy‐terminal domain (C‐domain) of CRT, resulting in the creation of a novel basic C‐terminus in place of the highly acidic C‐terminus of the wild type (WT) protein and a loss of the C‐terminal KDEL motif,^3^, ^4^ which is an endoplasmic reticulum (ER) retention sequence. This results in mutant CRT localization on the cell surface^14^, ^15^, ^16^ and the extracellular space via Golgi‐mediated secretion.^15^, ^17^, ^18^, ^19^

The mechanisms by which CRT mutants drive aberrant megakaryocyte differentiation into platelets and myelofibrosis are under intense investigation. In healthy individuals, megakaryocyte growth and differentiation are triggered by the growth factor thrombopoietin (TPO) which is released from the liver.^20^, ^21^ TPO binds to its receptor (MPL or TPOR), on the surface of megakaryocytes, which leads to structural rearrangements, dimerization and downstream activation of JAK–STAT signalling cascade.^22^, ^23^, ^24^ In MPNs, CRT mutants activate MPL signalling independently of TPO.^25^, ^26^ Binding of CRT mutants to MPL drives MPL dimerization^27^ and cytokine‐independent constitutive activation of the JAK–STAT pathway.^16^, ^25^, ^26^, ^27^, ^28^

The phenotypes of patients with ET caused by the *JAK2* V617F mutation or the *CALR* mutations are different. These include markedly increased platelet counts in ET patients with *CALR* mutations compared to those with *JAK2* mutations, but a relatively lower thrombotic risk.^3^, ^4^, ^29^ Based on the unique clinical aspects of mutant CRT‐mediated MPNs and the distinct interactions that trigger cell transformation, a better understanding of the role of mutated CRT in the pathogenesis of MPNs could lead to new diagnostics and treatments for MPN patients with *CALR* mutations.

Various mouse models have been developed which recapitulate several aspects of *CALR* mutation‐driven MPN disease.^26^, ^30^, ^31^, ^32^, ^33^, ^34^, ^35^, ^36^ These include an increase in the blood platelet counts (thrombocytosis) and megakaryocyte counts in the BM.^26^, ^30^, ^31^, ^32^, ^33^, ^34^, ^35^, ^36^ The expression of CRT_Del52_ in a homozygous context in mice drives more severe thrombocytosis when compared to either the heterozygous CRT_Del52_ mice^32^, ^34^ or the CRT_Ins5_ mice.^34^ MF was observed only in CRT_Del52_‐based mouse models^30^, ^32^, ^34^ but not in CRT_Ins5_ expressing mice.^30^, ^34^ Homozygous CRT_Del52_ mice show minor or mild fibrosis in the BM and spleen accompanied by splenomegaly, decrease in the BM cellularity, megakaryocyte hyperplasia and extramedullary hematopoiesis.^32^, ^34^ Heterozygous CRT_Del52_ knock‐in mice do not progress to MF, in contrast to MPN patients heterozygous for CRT_Del52_ who often develop MF.^32^, ^33^, ^34^ This could be explained by reduced activation of murine MPL signalling compared to human MPL activation by either murine or human CRT mutants.^33^, ^34^

Some studies have reported that mice expressing mutant CRT proteins exhibit the increased self‐renewal capacity of HSCs when compared to WT HSCs^30^, ^33^, ^34^ but this has not been observed in other studies.^31^, ^32^ The discrepancies between the different mouse models could be related to specific knock‐in constructs used or to the specific type of mouse model, which can affect the expression levels, the expression patterns, the relative functionality of the mutant CRT proteins, and whether or not endogenous calreticulin is also expressed. A recent study shows that CRT haploinsufficiency itself augments the self‐renewal activity of HSCs in mice^35^ which is of relevance to human disease, where the majority of *CALR* mutations occur in a heterozygous context.^3^, ^4^ Although, the different mouse models clearly substantiate the role of MPN‐linked CRT mutations as drivers of MPNs, there are differences not only between the different models but also between the MPN characteristics reported in mouse models compared to human MPNs. For instance, in contrast to the observations in mouse models,^30^, ^34^, ^36^ CRT_Ins5_ expressing ET patients have higher platelet counts compared to CRT_Del52_ ET patients.^37^, ^38^ The different mouse models that are described and their recapitulation of human disease has recently been extensively reviewed elsewhere^39^ which can be referred to for more detailed insights.

## 2 CALCIUM BINDING BY CALRETICULIN AND ITS RELEVANCE TO CANCER

CRT is a 46 kDa protein composed of a conserved globular N‐terminal lectin domain, a flexible proline‐rich polypeptide‐binding P‐loop, and a C‐terminal domain (Figure 1A). An important function of CRT is the buffering of calcium (Ca^2+^) ions in the ER, via multiple low affinity binding sites for Ca^2+^ ions,^40^, ^41^ which play a critical role in the maintenance of ER Ca^2+^ homeostasis. In addition to the high‐affinity Ca^2+^ binding site at D328 of the N‐domain (*K*_*D*_ ~ 20 μM) (Figure 1A), 4–6 low‐affinity, high‐capacity Ca^2+^ binding sites (*K*_*D*_ ~ 600 μM) were identified in the C‐terminal region of CRT.^41^ Ca^2+^ binding to these sites increases the structural stability of the C‐domain of CRT^41^ which can undergo Ca^2+^‐dependent unfolding or folding into an α‐helix and contact with the flexible P‐loop.^42^

Maintenance of Ca^2+^ levels in the ER via sequestration by Ca^2+^ binding proteins is important for many calcium‐dependent proteins and processes.^43^ Low calcium levels can lead to disruptions of homeostasis in the ER and activation of the unfolded protein response (UPR), leading to adjustments to protein folding in the ER to ensure cell survival.^44^ Although both the CRT_Del52_ and CRT_Ins5_ mutants have novel C‐terminal domains, a recent study by Ibarra et al. indicates that CRT_Ins5_ and CRT_Del52_ differ in their calcium binding properties and the downstream pathogenic effects.^36^ Compared with CRT_Ins5_, CRT_Del52_ is suggested to lose more of its calcium binding capacity due to the smaller number of acidic residues that are retained in the novel C‐domain of CRT_Del52_.^36^ Restoration of the calcium‐binding capacity in CRT_Del52_ cells via co‐expression of just the P + C domains of WT CRT decreased the survival of CRT_Del52_ cells, suggesting that CRT_Del52_ requires a low ER Ca^2+^ environment (high cytosolic calcium) to maintain cell survival.^36^ Megakaryocytes that express CRT_Del52_ display higher levels of Ca^2+^ mobilization into the cytosol compared to cells obtained from patients with a *JAK2* mutation or a CRT_Ins5_ mutation.^38^

Ibarra et al. further demonstrated that, compared to WT CRT and CRT_Ins5_ expressing cells, CRT_Del52_ expressing cells exhibited an upregulation of genes related to the inositol requiring‐enzyme 1 alpha (IRE1α)/X‐box‐binding protein 1(XBP1) UPR pathway. This, in turn, stimulated the expression of B‐cell lymphoma (BCL)‐2 protein,^36^ an anti‐apoptotic protein that prevents cell death and increases cell survival.^45^ It was also seen that CRT_Del52_ cells were able to sustain this IRE1α/XBP1 response by increasing the transcription of the *ITPR1* gene that encodes the Inositol‐1,4,5‐triphosphate (IP3R) receptor, one of the mediators of calcium efflux from the ER. CRT_Del52_ was thus able to maintain consistent depletion of Ca^2+^ levels in the ER,^36^ and a constitutive store‐activated Ca^2+^ entry (SOCE) response.^46^ These studies suggest an important distinction between CRT_Del52_ and CRT_Ins5_ expressing cells; CRT_Del52_ expressing cells promote cell survival via activation of UPR which is not seen in CRT_Ins5_ cells. Further research can be conducted to examine the distinct oncogenic pathways used by CRT_Del52_ and CRT_Ins5_, which could lead to therapeutics based on the specific mechanisms of pathogenesis.

Megakaryocytes from MPN patients with *CALR* mutations also display spontaneous calcium influx into the cytosol *via* the calcium release‐activated calcium channel protein 1 (ORAI1),^46^ residing in the plasma membrane (PM), which mediates the SOCE response.^47^ Upon Ca^2+^ depletion in the ER, the stromal interaction molecule 1 (STIM1), a calcium sensor located in the ER membrane, undergoes conformational changes, which weakens its interaction with ERp57,^48^ a thiol‐disulfide oxidoreductase. ERp57 is a co‐chaperone for WT CRT^49^ that is shown to be impaired in binding to CRT mutants.^50^ Dissociation of the complex between STIM1, ERp57 and CRT mutants in the ER, is suggested to result in enhanced STIM1 oligomerization and relocation to ER‐PM junctions, where it gates the ORAI1 channel,^47^ thus promoting enhanced SOCE activation, Ca^2+^ influx into the cytosol and cell proliferation.^46^ Inhibition of SOCE reduced the cytokine‐independent proliferation of mutant CRT‐expressing megakaryocytes.^46^

Overall, altered cellular calcium homeostasis is a component of the pathogenic effects of the CRT mutants, and some studies suggest that disruptions induced by CRT_Del52_ are more severe than those induced by CRT_Ins5_.

## 3 MUTANT CRT MULTIMERIZATION

Compared to WT CRT, CRT_Del52_ and CRT_Ins5_ have a high propensity to form homomultimers.^51^, ^52^ Cytokine‐independent MPL activation requires homomultimerization of CRT mutants which is dependent on the intermolecular interactions between mutant CRT monomers involving the novel C‐domains^51^, ^52^, ^53^ and/or the non‐mutated N‐domains.^52^, ^54^

The novel C‐domain cysteines (C400 and C404) along with the C163 residue in the N‐domain mediate the formation of disulfide‐linked dimers/multimers of CRT_Del52_.^52^ Alanine substitutions of these three cysteines (CRT_Del52_‐_3CA_) abrogated the disulfide‐linked dimerization of CRT_Del52_ and reduced its ability to bind MPL. However, the CRT_Del52_‐_3CA_ mutant exhibited only a small reduction in the MPL‐mediated cell proliferation Combination of CRT_Del52_‐_3CA_ with mutations of N‐domain residues, specifically, D165, D166 and H170, further reduced the dimer formation, accompanied by a significant impairment of MPL binding and cytokine‐independent proliferation.^52^ Oligomerization was also significantly decreased upon truncation of the novel C‐domain of CRT_Del52_, especially for CRT_Del52‐∆36_ in which 36 C‐terminal residues are deleted.^52^ Venkatesan et al. proposed a structural model in which CRT_Del52_ dimers are stabilized by ionic interactions between the N‐domains and disulfide bonds formed by C400 and C404 of the CRT_Del52_ monomers. The involvement of additional C‐terminal residues was not tested (Figure 1B).^52^

More recently, an extensive mutagenesis screen of the CRT N‐domain revealed that zinc (Zn^2+^) binding to three histidine residues (H99, H145 and H170) mediates homomultimerization and oncogenic activity of CRT_Del52_.^54^ CRT_Del52_ mutant lacking these three histidines lost Zn^2+^ binding ability and could not form homodimers and CRT_Del52_‐MPL heteromeric complexes. Furthermore, the application of zinc chelators abrogated JAK–STAT signalling.^54^ Examination of CRT dimers found in crystal structures suggests that Zn^2+^ binding sites might be formed by pairs of interacting residues, H99‐C163, H145‐D135 and H170‐D166, which are located in close proximity to the N‐domain dimerization interface identified by Venkatesan et al.^52^ (Figure 1B).

Another recently proposed model of CRT_Del52_ dimerization is based on experimental and computational results.^53^ The first 28 residues (367–394) of the CRT_Del52_ C‐tail were experimentally determined to be important for CRT_Del52_ and MPL dimerization as well as for cytokine‐independent cell proliferation.^53^ In this model, the two CRT_Del52_ monomers dimerize through coiled‐coil interactions between the positively charged C‐terminal α‐helices (residues 367–391) while the cysteines of the CRT_Del52_ C‐terminus are not involved in the formation of productive dimers.^53^ Additional structural studies are needed to fully uncover the molecular structures of functionally relevant dimers of CRT mutants. A model that could resolve some of the discrepancies is further discussed below.

## 4 MPL ACTIVATION AND SIGNALLING

**FIGURE 2:**
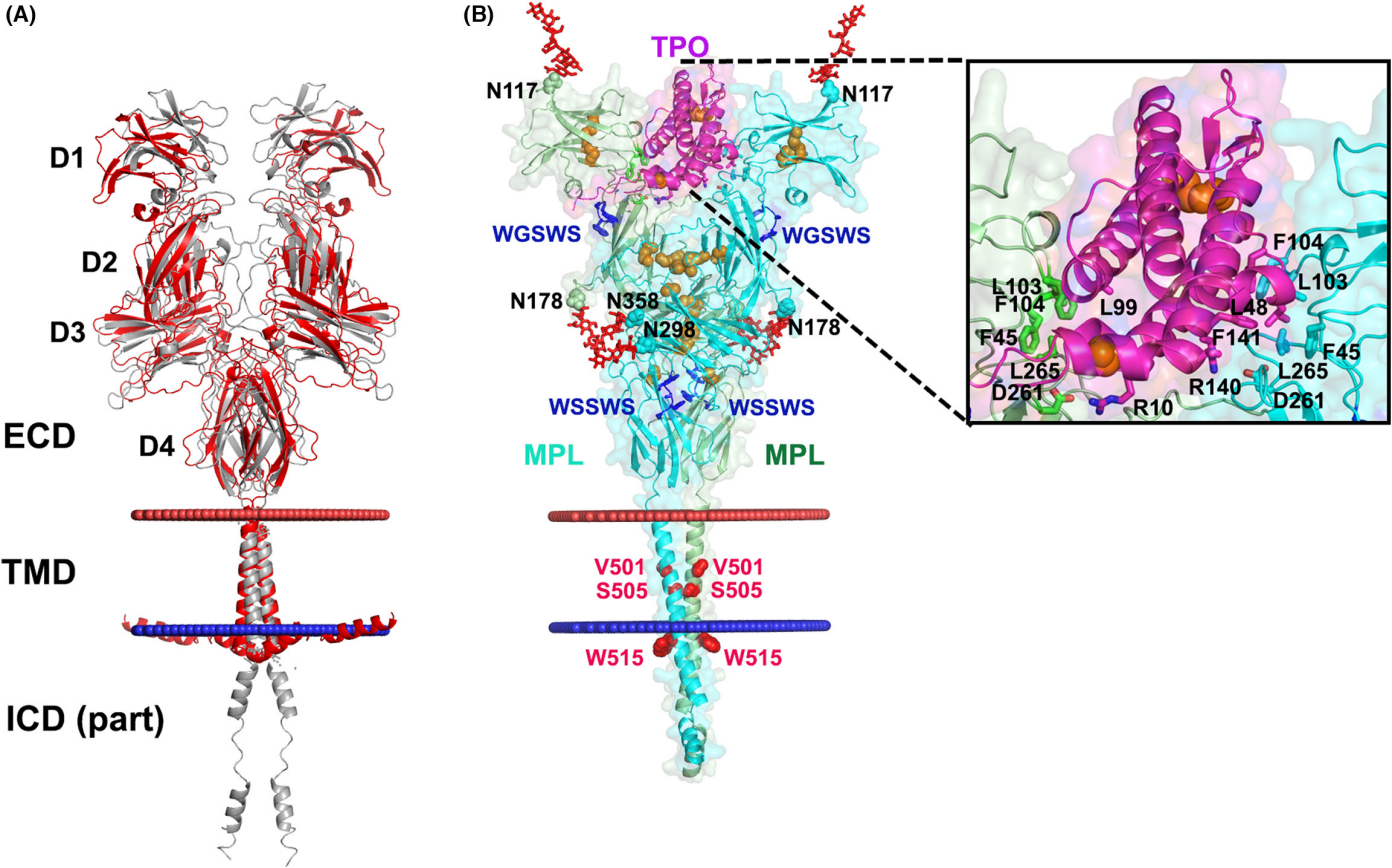
Structural models of human MPL dimers generated by AlphaFold‐multimer (AFM).^76^ (A) Superpositions of models of homodimers of human MPL (residues 26–525) demonstrate variable distances between the extracellular domains (ECD) of two MPL subunits. Models with closed and open spaces between D1‐D2 pairs of the two subunits are shown in grey and red cartoon representations, respectively. The conformations and spatial positions of the ICDs are variable. The positions of the membrane boundaries calculated using the PPM (Positioning of Proteins in Membranes) web server^92^ are shown as red (extracellular side) and blue (intracellular side) spheres. (B) A model of the TPO: MPL (1:2) heterotrimer generated by AFM (pLDDT 67.6, ptmscore 0.569 and iptm 0.523). The largest predicted alignment errors (PAE) are in the loops (residues 194–238, 319–327, 336–348), TM α‐helices and ICDs. The TM α‐helical dimer was generated by the TMDOCK server^93^ to substitute for the AFM‐generated TMD dimer. The TMDOCK‐modeled TMD dimer has a left‐handed helix arrangement with V501 and S505 at the dimerization interface, which corresponds to the active receptor state.^23^, ^58^ MPL subunits (coloured green and cyan) and TPO (coloured purple) are shown by cartoon and surface representations. N‐linked glycans attached to N117, N178, N298 and N358 of each MPL subunit are shown as red sticks. The WGSWS and WSSWS motif in D2 and D4, respectively, are shown by sticks coloured dark blue. Cysteine residues of MPL and TPO are shown as orange spheres. Mutated residues associated with the constitutive activation of MPL (V501, S505 and W515)^62^, ^63^, ^64^ are marked by red spheres. The inset in B shows residues of two MPL subunits (F45, E46, D47, L103, F104, D261 and L265) contacting two sides of the TPO four‐α‐bundle that are shown to be important for TPO binding and/or TPO‐induced MPL activation.^56^, ^57^

MPL is a cell surface class I cytokine receptor that regulates megakaryocyte differentiation and platelet production. MPL is synthesized and folded within the ER via the calreticulin/calnexin cycle. Human MPL contains 635 amino acid residues and three distinct domains: the extracellular domain (ECD) composed of two cytokine receptor homology modules (CRM1 and CRM2), each formed of adjacent pairs of fibronectin III‐like subdomains (D1–D2 and D3–D4, respectively), a single‐α‐helical transmembrane domain (TMD) and a mostly unstructured intracellular domain (ICD; Figure 2A; further discussed below). The membrane distal CRM1 acts as a brake on MPL activation and cell proliferation,^55^ and is required for TPO binding.^55^, ^56^ Mutations of residues located in the hinge between the D1 and D2 (F104, F45, L103, D261 and L265) impaired TPO binding and/or TPO‐dependent MPL activation (Figure 2B; further discussed below).^56^, ^57^ MPL activation is driven by dimerization of TMDs in a specific helical orientation with S505 at the contact interface.^23^, ^58^ In addition to the inactive and the physiologically active conformations of the TMD dimers, five other dimeric orientations of murine MPL transmembrane helices were identified, which induced cell proliferation and in vivo myeloproliferative disorders.^58^ A juxtamembrane motif ^514^KWQFP^518^ in the ICD of murine MPL regulates the transmembrane helix orientation and prevents receptor self‐activation.^58^, ^59^, ^60^ Furthermore, Box1 (^530^PSLPDL^535^) and Box2 (^566^SLLEILPKSSERTPL^580^) regions of the ICD are essential for binding of JAK2/TYK2 kinases and downstream signal transduction.^22^, ^61^

Fluorescence imaging of class I cytokine receptors in the plasma membrane at physiological densities demonstrated that MPL exists primarily in a monomeric form in resting cells and efficiently dimerizes upon ligand binding.^22^ Ligand binding to MPL and recruitment of JAK2 contribute additively to the stability of the TPO:MPL:JAK2 (1:2:2) signalling complex.^22^ Estimation of the energy contributions of different interactions indicates a low intrinsic dimerization affinity of MPL and JAK2 subunits, which increases in the JAK2 V617F and MPL W515L mutants. TPO binding provides a more significant energy contribution, thus shifting the equilibrium towards MPL dimerization.^22^ Constitutive activation of oncogenic MPL mutants, including W515X (X defines mutations to 17 amino acids, except Cys and Pro),^62^ V501A^63^ and S505N,^64^ is a consequence of the receptor being maintained in the active dimeric state even in the absence of ligand.^60^

**FIGURE 3:**
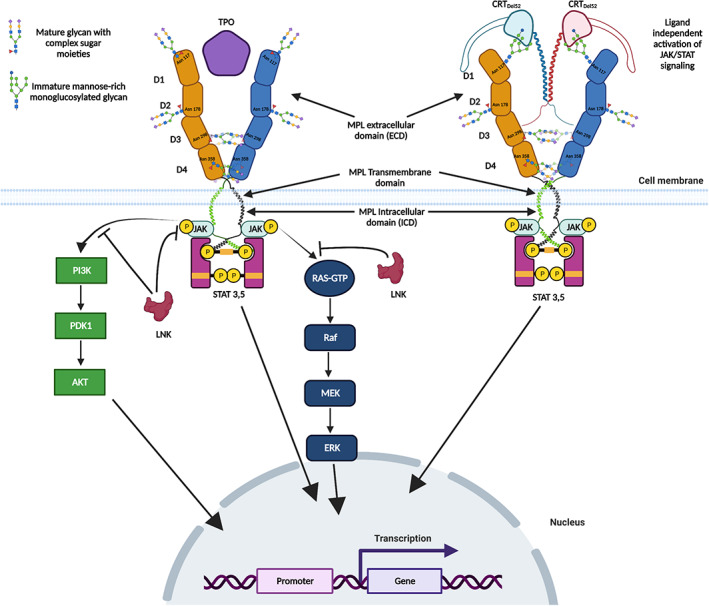
TPO or mutant CRT‐induced activation of MPL signalling. Left panel: TPO binds to MPL present on the surface of megakaryocytes and HSCs and triggers conformational changes and dimerization of the MPL receptors that results in the recruitment and dimerization of the JAK2 tyrosine kinase, which, in turn, *trans*‐phosphorylates the TK domains of the JAK2 subunits and tyrosine residues in the ICDs of MPL subunits. This triggers the recruitment of STAT proteins (STAT3 and STAT5) to phosphorylated tyrosines on the ICDs of MPL, followed by phosphorylation, dimerization and translocation of the STAT proteins to the nucleus where they regulate gene expression. TPO binding to MPL and the consequent activation of the JAK2 TK domain also activates the PI3K/AKT and the MAPK/ERK signalling pathways. MPL signalling activated by TPO is inhibited by adaptor proteins such as LNK. Right panel: MPN‐linked CRT mutants mediate TPO‐independent activation of MPL signalling via the JAK–STAT pathway. The interactions involve an immature glycan on MPL that binds to the N‐glycan‐binding site of mutant CRT as well as MPL interactions with the novel positively charged C‐domain of mutant CRT. The figure was prepared using BioRender (BioRender.com).

MPL dimerization brings together the two JAK2 proteins that are bound to the two MPL ICDs.^22^ In addition to JAK2, TYK2 is also involved in MPL‐mediated signalling, although at very high TPO concentrations.^61^ JAK2 dimers are formed through interactions between the pseudokinase (PK) domains,^22^ as in JAK1 dimers (PDB ID: 7T6F).^65^ JAK2 dimerization induces the *trans*‐phosphorylation of each JAK2 protein at Y1007 and Y1008 in the activation loop of the tyrosine kinase (TK) domain leading to TK activation.^66^ Activated TK subsequently phosphorylates specific tyrosine residues in the associated MPL subunits (Y626 and Y631)^67^ that act as docking sites for SH2 domain‐containing adaptor proteins.^24^ The next step in signalling involves the recruitment of the signal transducers and activators of transcription (STAT3 and STAT5) to the phosphorylated receptor tyrosines. Upon phosphorylation by JAK2, STATs dimerize and translocate to the nucleus to drive cytokine‐dependent gene expression (Figure 3).^68^ TPO‐induced MPL activation also stimulates signalling pathways associated with mitogen‐activated protein kinases (MAPK),^69^ and phosphatidyl inositol 3‐kinase (PI3K).^70^

Negative regulation of MPL signalling depends on the expression of cytokine signalling suppressor proteins. Lymphocyte adaptor protein, LNK, negatively regulates TPO‐induced STAT3 and STAT5 phosphorylation and MAPK activation, thus inhibiting growth, endomitosis and proliferation of megakaryocytes,^71^ as well as HSC self‐renewal and quiescence by binding to phosphorylated JAK2.^72^ Internalization and degradation of receptor‐ligand complexes also contribute to the negative regulation of MPL signalling. MPL internalization is mediated by clathrin‐dependent endocytosis and requires the Y626 residue and two dileucine motifs in the Box2 of the ICD (^567^LLEIL^571^).^73^ Furthermore, the ^591^YRRL^594^ motif in the ICD of MPL is involved in clathrin‐dependent endocytosis via the adaptor protein AP2, while the ^521^YRRL^524^ motif participates in the lysosomal targeting of MPL.^74^ MPL is also degraded by the proteasomal pathway following ubiquitination at K553 and K573 residues by the E3 ubiquitin ligase c‐Cbl.^75^

In the absence of an experimental atomic‐level structure of the full‐length MPL, computational modelling provides an alternative to assist structure‐based studies and for illustrative purposes. The AlphaFold‐Multimer (AFM), an artificial intelligence‐based computational tool that outperformed other approaches in structural prediction of protein complexes,^76^ was applied to model complexes of human MPL. AFM‐generated dimers demonstrate two subsets of conformations: those with closed and open ligand binding sites between the D1‐D2 subdomains of the two ECDs (Figure 2A). Modelling of TPO:MPL (1:2) trimeric complex with AFM resulted in an open (presumably active) dimer conformation stabilized by TPO bound between two ECDs, forming contacts with residues at the D1‐D2 interface that have been implicated in TPO binding and MPL activation^56^, ^57^ (Figure 2B, inset).

## 5 MUTANT CRT‐MPL COMPLEXES AND DYSREGULATED MPL SIGNALLING IN MPNS

CRT_Del52_ and CRT_Ins5_ induce cytokine‐independent cell proliferation and oncogenesis via specific interaction with MPL,^16^, ^25^, ^26^ though transient weak signalling via granulocyte colony‐stimulating factor receptor (G‐CSFR) has been also reported.^25^ WT CRT forms transient complexes with the monoglucosylated N‐linked glycans of MPL in the ER as it does with other N‐glycosylated proteins.^7^ Conversely, CRT mutants form stable complexes with MPL via elaborate interactions involving the residues in the N‐domain and novel C‐domain of CRT mutants,^16^, ^26^, ^28^, ^52^, ^53^ in addition to the glycan‐driven interactions.^14^, ^27^, ^28^

Substitution of Y109^14^, ^27^ and D135^14^, ^27^, ^28^ residues in the glycan binding site resulted in the loss of interactions of CRT mutants with MPL^14^, ^27^, ^28^ and their inability to support cytokine‐independent STAT5 transcriptional activity^25^ and cell proliferation.^14^, ^28^ Moreover, the novel C‐domain of CRT mutants is critical for binding and activation of MPL.^28^, ^52^, ^53^ Particularly important are the residues 376–392 of CRT_Del52_,^52^, ^53^ as the deletion of 28 (∆28) and 36(∆36), but not of 19 (∆19) residues from the CRT_Del52_ C‐terminus reduced the ability of CRT_Del52_ to induce cytokine‐independent proliferation of MPL‐expressing cells.^52^

The ECD of MPL has four N‐glycosylation sites, N117, N178, N298 and N358. Removal of N117 site alone, as well as the mutations of any three MPL glycosylation sites, lowered the surface expression of MPL and TPO‐dependent cell proliferation.^77^ N117 is essential for activation of MPL signalling by CRT_Del52_ and CRT_Ins5_, while N178 supports weak MPL activation by CRT_Ins5_.^25^ CRT mutants determine the transport of partially immature MPL (with N117‐linked mannose‐rich glycan^27^) from ER to the cell surface via the secretory pathway.^25^, ^27^ When complexes of MPL with CRT mutants are trafficked through the Golgi apparatus, N178, N298 and N358 achieve mature glycosylation, whereas N117 is protected from maturation by binding to mutant CRT.^27^ CRT mutants also rescue traffic to the cell surface and activation of traffic‐defective MPLs, including K39N, R102P, G509N mutants and constructs carrying ER‐retention signals.^27^ Although MPL and mutant CRTs become engaged in the ER, surface localization of the mutant CRT‐MPL complex is indispensable for the activation of MPL signalling.^14^

CRT_Del52_ interacts with D1 of MPL not only by binding the N117‐linked glycan but also by forming ionic interactions between the basic residues in the CRT_Del52_ C‐tail (residues 378–391) and negatively charged patches centred at ^44^TFED^47^ and ^52^WDEE^55^ in MPL ECDs.^53^ A patch of 8 hydrophobic (^104^FFPLHLWV) residues was identified in the hinge between D1‐D2 subdomains of MPL that is required for MPL activation by CRT mutants but not for binding.^27^

CRT mutants are also detected in the plasma of MPN patients.^15^, ^19^, ^52^ A recent study shows that the secreted forms of CRT mutants are stabilized by binding to the soluble transferrin receptor 1 (sTFRC).^15^ Paracrine activation of MPL signalling by secreted forms of CRT mutants was not observed when healthy cells expressing MPL and WT CRT were treated with supernatants from CRT_Del52_ expressing cells.^18^ However, a recent study demonstrates that exogenous recombinant CRT_Del52_ promotes cytokine‐independent proliferation, particularly of cells expressing both mutant CRT and MPL.^15^ Cells expressing both MPL and mutant CRTs expose immature MPL glycans on the cell surface to induce additional activation by exogenously supplied CRT mutants.^15^

**FIGURE 4:**
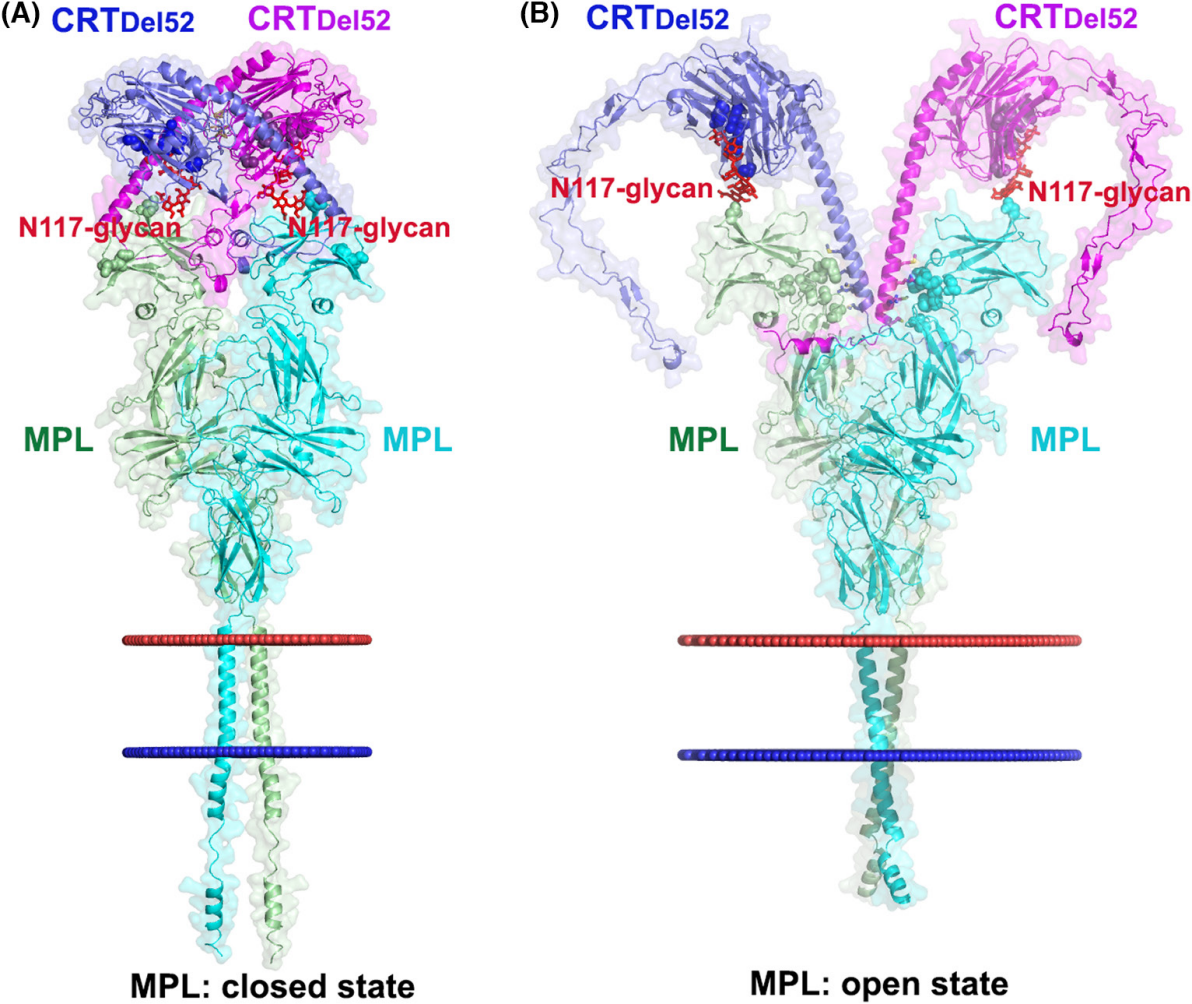
Hypothetical models for MPL activation by CRT_Del52_. (A) Docking of a CRT_Del52_ dimer (from Figure 1B) onto the AFM‐generated model of a MPL dimer in the closed conformation. (B) Docking of two CRT_Del52_ molecules into the ligand binding pocket of the AFM‐generated model of the MPL dimer in an open conformation (from Figure 2B). Side view of a CRT_Del52_:MPL (2:2) heterotetramer is shown. Subunits of MPL (coloured green and cyan) and CRT_Del52_ (coloured blue and purple) are shown as cartoon and surface representations. N‐glycans linked to N117 of MPL subunits are shown as red sticks, CRT_Del52_ residues in the glycan binding pocket of both molecules are shown as blue and purple spheres. In (B), the green and cyan spheres indicate residues whose mutations affect TPO and/or CRT_Del52_ binding and/or MPL activation (F45, E46, D47, L103, F104, D261 and L265).^53^, ^56^, ^57^ Residues of CRT_Del52_ (M380, R384, M387, R388, M391) predicted to interact with the indicated MPL residues are shown as blue and purple sticks for different subunits. The positions of membrane boundaries calculated by the PPM web server^92^ are shown as red (extracellular side) and blue (intracellular side) spheres.

Despite the accumulation of a vast amount of experimental data supporting the formation of MPL‐CRT complexes, the exact mode of binding of CRT mutants to MPL and the molecular mechanism of mutant CRT‐induced MPL activation remains to be characterized. Modelling with AFM allowed us to construct tentative structural models for different conformations of the CRT_Del52_:MPL (2:2) heterotetrameric complex (Figure 4) that has been suggested based on size exclusion chromatography data.^27^ The previously proposed CRT_Del52_ dimer (Figure 1B) can be unambiguously docked onto the AFM‐generated closed conformations of MPL dimers due to the matching of distances between N117 of two MPL subunits and between K111 residues in the two glycan binding sites of the CRT_Del52_ dimer (Figure 4A). In this model, the small α‐helical fragments (residues 400–406) of the CRT_Del52_ C‐tail can interact with hydrophobic residues from the MPL ligand‐binding pocket, while basic C‐tail residues from the ^385^RKMR^388^ fragment are in close proximity to residues ^52^WDEE^55^ forming a negative patch in MPL implicated in the binding of mutant CRT.^53^ However, the CRT_Del52_ dimer appears to be incompatible with the open state of the MPL dimer, where the distance between the two N117 residues is significantly increased. Docking of CRT_Del52_ into the open conformation of the MPL dimer (Figure 4B) requires dissociation of the CRT_Del52_ dimer, rotation of N‐domains with the bound N117‐glycans, re‐positioning of C‐domains to form hydrophobic contacts between C‐tail methionines and the ^103^LFF fragment involved in receptor activation^27^ and ionic interactions between basic C‐tail residues and acidic MPL residues, including E46 from the negative patch.^53^ In this model, most interactions occur between D1 residues of MPL and residues from the ^376^RMRRMRRTRRKMRRKM^391^ fragment of CRT_Del52_ that are shown to be important for MPL activation.^28^, ^52^, ^53^ In either, the open or closed conformations of MPL dimers, both N117‐linked glycans have proper interactions with residues forming the CRT glycan binding pockets.

Based on these structural models, a 2‐step activation of MPL by CRT mutant can be proposed. In the first step, the CRT_Del52_ dimer binds and stabilizes the MPL dimer in the closed conformation of ECDs mainly due to interactions between N‐domains and MPL N117‐linked glycans (Figure 4A). In the second step, MPL subunits may rearrange due to the structural plasticity of MPL dimers. Separation of two MPL ECDs would facilitate the dissociation of weakly interacting N‐domains of CRT_Del52_ dimer followed by the binding of C‐domains into the MPL ligand‐binding site while maintaining interactions with N117‐linked glycans. The binding of CRT_Del52_ C‐terminal α‐helices additionally stabilizes MPL dimers in the open conformation of ECDs (Figure 4B). This ECD conformation initiates a rearrangement of TM α‐helices into the left‐handed active dimeric state, which, in turn, causes the repositioning of ICDs interacting with JAK2 molecules, and consequently promotes JAK2 dimerization.

The model of CRT_Del52_ docked in the open conformation of MPL (Figure 4B) closely resembles the model recently proposed by Constantinescu and coauthors (figure 6 in Ref. 53).^53^ In the latter model, the C‐terminal α‐helices of CRT_Del52_ form a left‐handed dimer that binds and stabilizes the MPL dimer via N117‐glycan‐driven interactions and electrostatic interactions between positively charged R279, K386 and K390 of CRT_Del52_ and negatively charged patches centred at ^44^TFED^47^ of both MPL subunits.^53^ However, the relative arrangements of C‐terminal α‐helices of CRT_Del52_ and the set of basic residues interacting with MPL are different in the two models. Additional studies are needed for the experimental validation of these models.

## 6 MHC CLASS I ANTIGEN PRESENTATION AND T CELL ACTIVATION BY MUTANT CRT IN MPN

**FIGURE 5:**
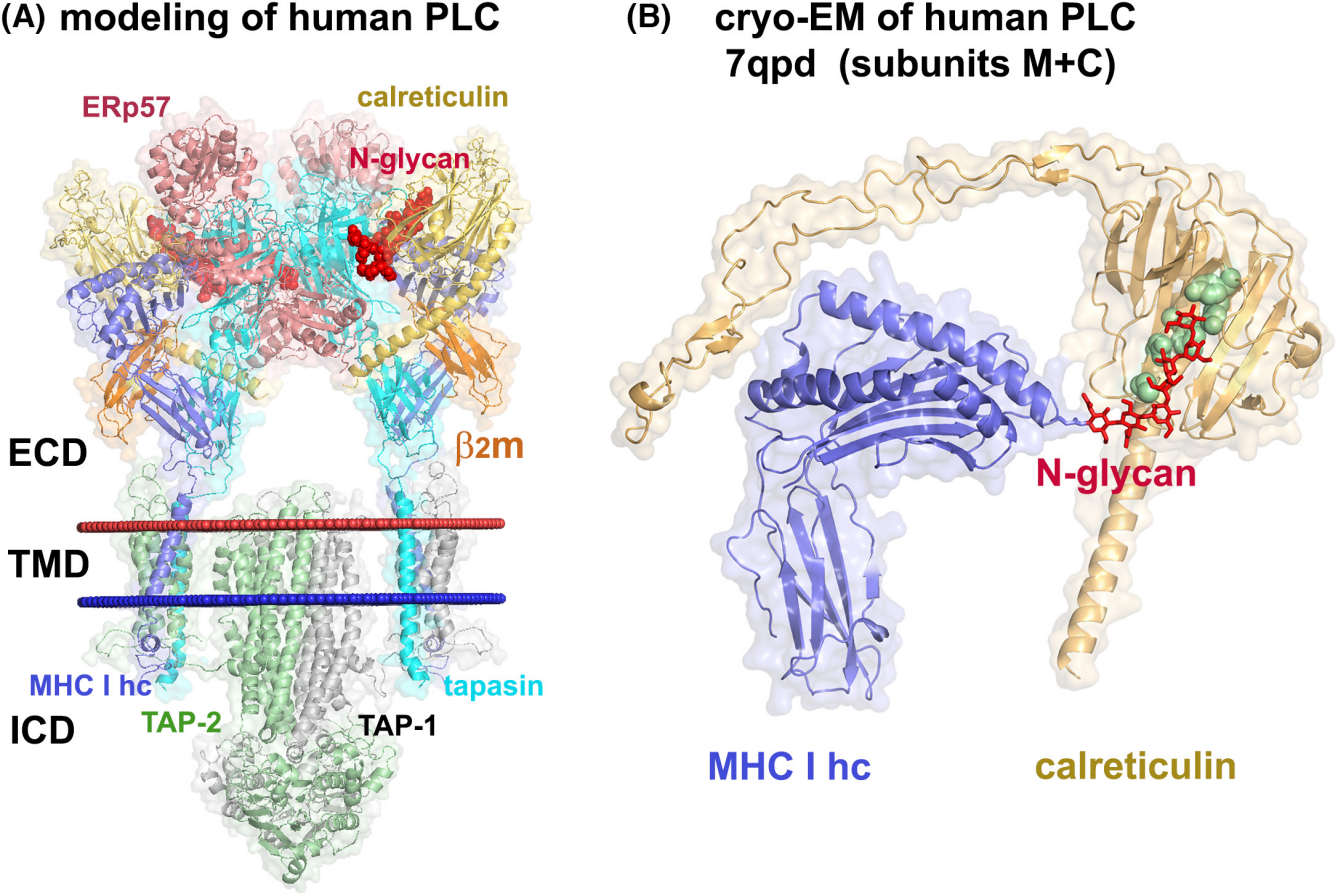
Human PLC components based on cryo‐EM images. (A) A cryo‐EM‐based model of the full‐length human PLC formed by two editing modules composed of 5 proteins each that are centred around the heterodimeric peptide transporter TAP1‐TAP2 composed of 20 TM helices and cytosolic nucleotide‐binding domains.^79^, ^81^ Molecules from the complex are shown as cartoon and surface representations coloured by the molecule type. N‐glycans linked to N86 of MHC class I heavy chain and N233 of tapasin are shown as red spheres. Positions of membrane boundaries calculated by the PPM web server^92^ are shown as red (extracellular side) and blue (intracellular side) spheres. (B) Interactions of N‐linked glycan (shown by red sticks) attached to N86 of MHC class I heavy chain (coloured blue) with calreticulin (coloured yellow). MHC class I heavy chain and calreticulin molecules that were extracted from the cryo‐EM structure of the human PLC (PDB ID: 7qpd, subunits M and C)^80^ are shown by cartoon and surface representations coloured blue and yellow, respectively. Residues from the glycan binding pocket of calreticulin^54^ are shown as green spheres.

CRT is a component of a membrane‐bound multi‐protein machinery in the ER called the MHC class I peptide loading complex (PLC).^78^ The PLC assists in the assembly of MHC class I molecules with peptides to allow CD8^+^ T cells to distinguish healthy and compromised cells. Within the PLC, there are two “editing” modules, each containing a peptide‐free MHC class I heavy chain in complex with β2‐microglobulin (β2m), surrounded by CRT, ERp57 and tapasin. Tapasin links the modules to the transporter associated with antigen processing (TAP1‐TAP2) subunits.^78^, ^79^, ^80^, ^81^, ^82^ A low‐resolution cryo‐EM‐based model of the PLC has been obtained (Figure 5A)^79^ and recently used to construct and simulate all‐atom molecular dynamics of the complete human PLC.^81^ Additionally, structures of water‐soluble domains of an editing module of the human PLC have been generated based on cryo‐EM images at 3.7 Å resolution.^80^ These structures reveal that CRT's glycan binding site engages a conserved MHC class I glycan at N86 of the MHC class I heavy chain (Figure 5B), while the C‐domain of CRT interacts with tapasin. Additionally, the tip of CRT's P‐domain interacts with ERp57 (Figure 5A).

Ectopic expression of mutant CRT proteins in CRT‐deficient cell lines resulted in significantly reduced MHC class I induction compared to cell lines in which WT CRT expression was restored.^17^ Forced ER retention of mutant CRT via the addition of a KDEL sequence at the C‐terminal end failed to rescue surface MHC class I levels. However, the addition of an ER retention motif rescued mutant CRT protein levels in the ER and partially restored the interactions of CRT mutants with tapasin. Reduced surface MHC class I levels combined with findings of low thermal stability of surface MHC class I complexes in cells expressing CRT mutants with or without the KDEL motif indicates suboptimal MHC class I assembly when mutant CRTs were expressed.^17^ These findings suggest that MPN‐linked CRT mutants are functionally impaired in facilitating MHC class I assembly, beyond their low ER availability.

CD4^+^ or CD8^+^ T cells are activated via the recognition of antigenic peptides presented in complexes with MHC class II or class I molecules, respectively. In cancers, the antigenic peptides are generally derived from mutated self‐proteins (tumour‐specific antigens or neoantigens). Activation of CD4^+^ T cell‐mediated immune responses against mutant CRT‐derived peptides in MPN was more frequently observed in MPN patients with ET compared to MF patients.^83^ Immune checkpoint blockade using anti‐PD1 and anti‐CTLA4 monoclonal antibodies induced mutant CRT‐specific T cell responses in blood samples from MPN patients, indicating that PD1 and CTLA4 expression suppress MPN‐specific T cell responses (both CD4^+^ or CD8^+^).^84^ Additionally, secreted forms of MPN‐linked CRT mutants suppress phagocytic uptake of dying cancer cells by immune cells and, thus, inhibit priming of CD8^+^ T cells.^19^, ^85^ MPN patients with activated JAK–STAT responses also show a high prevalence of platelet‐CD8^+^ T cells aggregates. Such aggregates reduced the cytotoxic potential of CD8^+^ T cells, indicating that high platelet counts could cause T cell immune suppression in MPN patients.^86^ These studies demonstrate that CRT mutant‐expressing cells have evolved mechanisms for evading the activation of protective immune responses in MPN patients to ensure successful tumorigenesis.

The MHC class I heavy chain locus is highly polymorphic,^87^ and the polymorphisms influence their peptide binding specificities. MHC class I allele representation in MPN patients from US and Danish cohorts was biased towards allotypes exhibiting weak predicted binding affinities to mutant CRT‐derived peptide sequences.^88^ HLA‐B*51:01, predicted to bind poorly to mutant CRT peptides, was found to be overrepresented. On the other hand, six MHC class I alleles, HLA‐A*11:01, HLA‐B*08:01, HLA‐B*44:02, HLA‐C*07:01, HLA‐C*07:02 and HLA‐C*06:02, predicted as strong binders of mutant CRT‐derived peptides were underrepresented in both cohorts.^88^ MHC class I skewing was specifically observed in CRT‐mutated MPNs. Pre‐stimulation of healthy donor PBMCs or pre‐immunization of mice with modified peptide sequences (heteroclitic peptides) could induce immune activation to an otherwise weak binding peptide.^88^ Thus, optimization of peptide sequences to allow presentation by overrepresented MHC alleles might provide an opportunity to activate mutant CRT‐specific T cell responses in MPN patients.

A peptide vaccine (CALRLong36) based on the common mutated C‐terminal amino acid residues found in MPN‐linked mutant CRT proteins was recently tested in phase I clinical trials.^89^ The patients were administered 15 vaccines over a course of one year. ET patients were found to respond better than MF patients. Six out of eight patients showed exclusive activation of CD4^+^ T cell responses, while one showed exclusive activation of CD8^+^ T cell responses that prevailed after the completion of the vaccination course. One patient showed activation of both CD4^+^ and CD8^+^ T cell responses.^89^

Taken together, these findings indicate negative effects of mutant CRT on MHC class I expression and T cell responses, but despite these effects, MPN CRT‐specific T cell responses are detectable in some MPN patients, particularly under the conditions of checkpoint blockade^84^ or following vaccination.^89^ Furthermore, the findings of MHC class I skewing in CRT mutated MPN patients^88^ suggest that antigen presentation via MHC class I helps control MPN, likely to a subclinical level.

## 7 CONCLUSIONS AND FUTURE PERSPECTIVE

This review summarizes current knowledge about somatic frameshift mutations of a multifunctional chaperone protein, CRT, that is able to both directly transform cells and evade immune responses. Stimulation of the MPL‐dependent JAK–STAT signalling pathway by CRT mutants is undoubtedly a key mechanism driving aberrant megakaryocyte growth, platelet production and myelofibrosis in ET and MF patients carrying *CALR* mutations. Understanding the structural details of complexes formed between CRT mutants, MPL and JAK2 proteins will allow for the design of inhibitors that could specifically target these complexes. Moreover, MPN‐linked CRT mutants alter cellular calcium homeostasis, which likely contributes to cellular survival and proliferative advantages. Uncovering the differential survival mechanisms activated by CRT_Ins5_‐like and CRT_Del52_‐like mutants might assist in the discovery of specific therapeutic targets for patients with different CRT mutations. Finally, mutant CRT has deficiencies in mediating MHC class I assembly, which is a possible mechanism of immune evasion in patients. Nonetheless, the measurements of memory T cell responses in patients treated with mutated CRT sequence‐based vaccines highlights the potential for immunotherapeutic targeting of MPN.

## AUTHOR CONTRIBUTIONS

**Harini Desikan:** Conceptualization (supporting); writing – original draft (equal); writing – review and editing (equal). **Amanpreet Kaur:** Conceptualization (supporting); visualization (supporting); writing – original draft (equal); writing – review and editing (equal). **Irina D Pogozheva:** Funding acquisition (supporting); software (lead); visualization (lead); writing – original draft (lead); writing – review and editing (lead). **Malini Raghavan:** Conceptualization (lead); funding acquisition (lead); supervision (lead); writing – review and editing (equal).

## CONFLICT OF INTEREST STATEMENT

The authors confirm that there are no conflicts of interest.
